# Innovative modified-net architecture: enhanced segmentation of deep vein thrombosis

**DOI:** 10.1038/s41598-024-81703-5

**Published:** 2024-12-28

**Authors:** Pavihaa Lakshmi B., Vidhya S.

**Affiliations:** School of Electronics Engineering, Vellore Institute of Technology, Vellore, 632014 Tamilnadu India

**Keywords:** Health care, Medical imaging

## Abstract

A new era for diagnosing and treating Deep Vein Thrombosis (DVT) relies on precise segmentation from medical images. Our research introduces a novel algorithm, the Modified-Net architecture, which integrates a broad spectrum of architectural components tailored to detect the intricate patterns and variances in DVT imaging data. Our work integrates advanced components such as dilated convolutions for larger receptive fields, spatial pyramid pooling for context, residual and inception blocks for multiscale feature extraction, and attention mechanisms for highlighting key features. Our framework enhances precision of DVT region identification, attaining an accuracy of 98.92%, with a loss of 0.0269. The model also validates sensitivity 96.55%, specificity 96.70%, precision 98.61%, dice 97.48% and Intersection over Union (IoU) 95.10% offering valuable insights into DVT segmentation. Our framework significantly improves segmentation performance over traditional methods such as Convolutional Neural Network , Sequential, U-Net, Schematic. The management of DVT can be improved through enhanced segmentation techniques, which can improve clinical observation, treatment planning, and ultimately patient outcomes.

## Introduction

Deep vein thrombosis (DVT) is a blood clot that forms in the deep vein of our calf muscle region in the lower extremities, causing leg pain or swelling. In rare cases, it may also occur in the arms. If the clot travels through the bloodstream, blocking blood flow in the lungs, it can lead to Pulmonary Embolism (PE)^1^. Emerging techniques in medical imaging, such as Deep Learning (DL), Machine Learning (ML), and Artificial Intelligence (AI), are revolutionizing the diagnosis of conditions like tumors, clots, cancer, etc., using different imaging modalities such as, Duplex Ultrasonography, Computed Tomography (CT), Magnetic Resonance Imaging (MRI) and Venography. DL uses neural networks to recognize patterns and features in medical images, enhancing efficiency and accuracy. ML uses algorithmic learning to discern patterns from historical data, aiding in the identification of thrombosis in deep veins. The integration of AI technologies further enhances the capabilities of image segmentation, allowing for analysis and interpretation of distinct features in medical images. In the realm of medical imaging, emerging techniques in image segmentation play a pivotal role. These advancements contribute significantly to accurate diagnosis and effective treatment planning across various medical fields. Especially, focusing on DVT segmentation techniques help in the accurate identification and delineation of affected areas, improving the performance in a more effective manner and early diagnosis. By segmenting relevant structures, clinicians can obtain valuable insights for timely diagnosis, enhancing patient outcomes. Qureshi, Imran, et al. investigated the benefits, potential drawbacks and future prospects of existing architectures in the field of medical image segmentation for diagnosing possible diseases^2^. Image segmentation is crucial for detecting different types of disease and lesion regions. It also introduces standard image segmentation algorithms and U-Net, examining their clinical applications^3^.

### Hierarchy of epidemiological risks

**Fig. 1:**
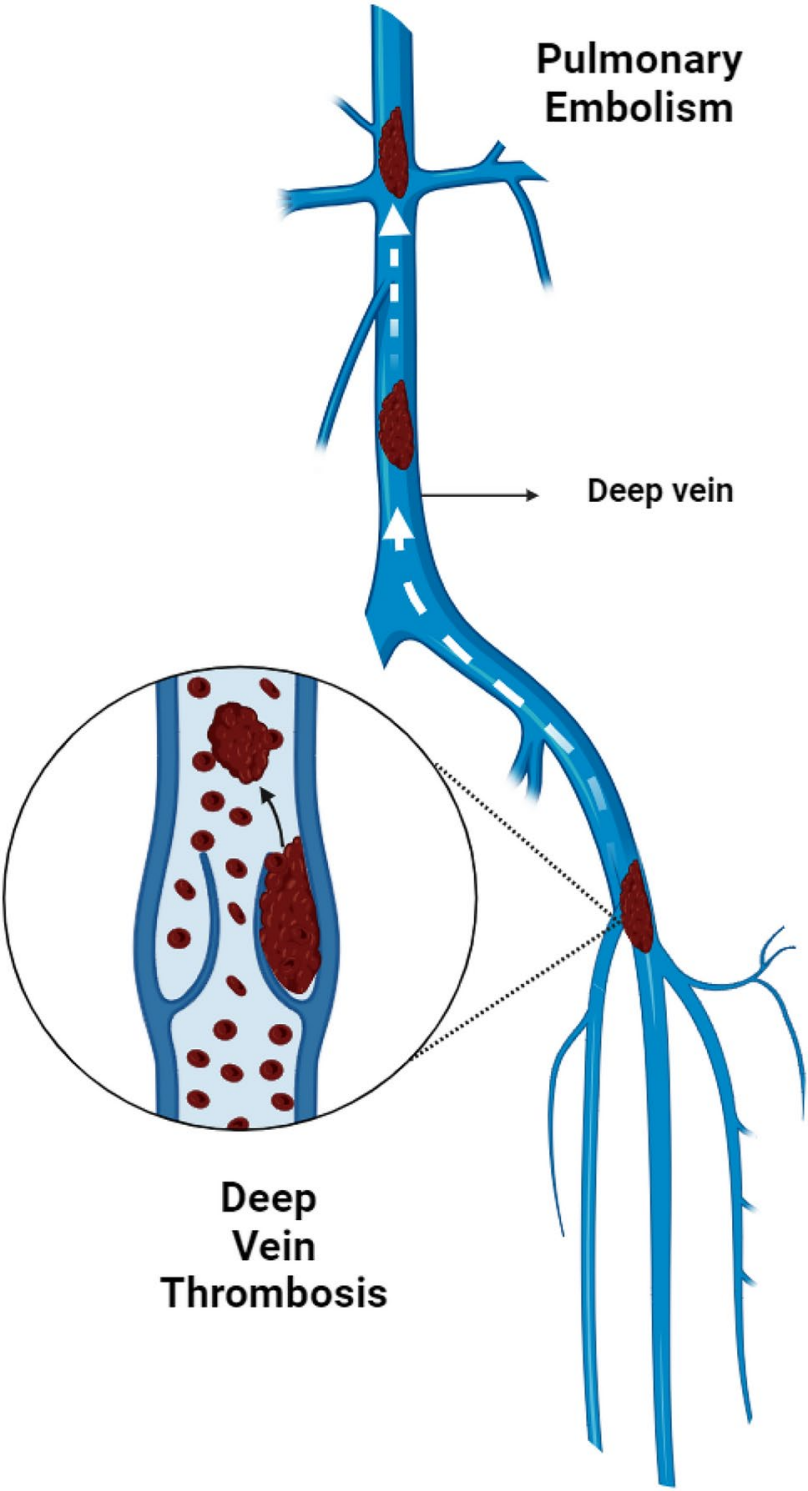
Anatomical representation of DVT occurrence and PE development.

DVT is a serious and dangerous condition that needs immediate care to avoid its blocking and risky state. DVT often affects the lower limb, where a clot forms in a deep vein of the calf muscle region. Figure 1 shows a visual representation of DVT and PE. It is a common venous thromboembolic (VTE) disorder with an incidence of 1.6 per 1000 annually. DVT is a significant medical concern, part of VTE disorders, and the third leading cause of cardiovascular disease-related deaths. DVT and PE are often undiagnosed due to their silent nature, leading to an underestimated incidence and prevalence. DVT’s annual incidence is around 80 cases per 100,000, with a prevalence of 1 case per 1000 for lower limb DVT. In the U.S., over 200,000 people develop venous thrombosis annually, and 50,000 cases are complicated by PE^4^.

### Clinical diagnosis and assessment of DVT patients

Zhang, Zejun, et al. investigated the history of DVT diagnostic procedures, early diagnosis using point of care equipment, the current state of diagnostic equipment, performance measurements, and emerging trends in detection methods, highlighting issues for improvement^5^. Shaziya Humera, and K. Shyamala compared CT images of lungs using Convolutional Neural Network (CNN) and U-Net models, finding U-Net outperforms CNN in lung field segmentation, highlighting DL’s potential in image recognition^6^. The study proposed a fully automatic method for identifying DVT using DL and Contrast Enhanced-MRI images. The approach was evaluated on 58 people who had recently been diagnosed with DVT. CNN outperformed other DL models, with a median Dice Similarity Coefficient (DSC) of 0.74 ± 0.17^7^. Hwang Jung Han, et al. compared the classification of DVT using CNN and ML algorithms on 659 participants. ML included logistic regression, SVM, RF, and extreme gradient boosts, while CNN-based models like VGG16, VGG19, Resnet50, and Resnet152 were evaluated. CNN models, particularly via the VGG16 model, classified DVT more effectively and accurately^8^. Liu Shing-Hong, et al. used a 2D CNN to evaluate the quality of light reflection rheography (LRR) signals and classify positive or negative DVT with high reliability. The LRR technique is used to assess DVT risks, and DVT is classified using a 2D CNN after signal quality is assessed. The study aimed to develop a wearable device using the LRR technique, which could help people with hidden DVT risks be examined for embolus formation. The accuracy of determining LRR signal quality was higher than the previous studies^9^.

This study demonstrates early-stage DVT segmentation and diagnosis using clinical data, starting with pre-processing, training, testing, and comparative analysis of classifiers using performance measures. The entire work is categorised as follows: Section 1 provides a detailed explanation of the DVT cause, sign and prevalence of risk factors, along with monitoring and identifying the DVT techniques. Section 2 focuses on analysing image processing techniques and addressing significant limitations. A detailed description of our proposed framework with a schematic block diagram and algorithm is explained in section 3. Section 4 discusses the performance of our model and comparative summary of existing techniques. Furthermore, it is concluded with discussion and the future scope in sections 5 and 6.

## Technical background

Schraut Jaiden Xuan, et al. developed a multi-output network using U-Net for segmentation and a CNN for classification. The model improved simultaneous classification results, achieving 97.72% accuracy and a 0.9691 dice coefficient^10^. The study introduced a deep multiscale convolutional neural networks for image segmentation. This approach was composed of three phases: an encoder, a U-net, and a decoder. The encoder was responsible for feature extraction from 2D image slices, and these features were then cascaded via deconvolution in the decoder by the U-net. The results showed improved accuracy and robustness in segmentation^11^. The Transformer-based Attention-Guided Network improved semantic segmentation in medical images by learning non-local interactions among encoder features, generating discriminative features, and reducing fine detail loss^12^.

### Immersive segmentation techniques

The Multi-scale Attention Net (MA-Net) is a neural network technique that integrated two blocks: the Position-wise Attention Block (PAB) and the Multi-scale Fusion Attention Block (MFAB). It improved performance under shadow, dynamic background, and illumination challenges. This showed potential for background-foreground segmentation in computer vision tasks^13^. In medical imaging research, DL play a crucial role by facilitating identification and segmentation, morphology, classification, and disease recognition. The development of U-Net architecture for internal organ area segmentation was examined by Krithika alias AnbuDevi M and K. Suganthi, with a focus on specific segmentation and performance metrics. GAN and U-Net could be cascaded for effective image synthesis^14^.

A DL-based automatic segmentation model was designed based on multiscale input and encoding-decoding technique. This model effectively extracted global and local images for segmentation tasks. As a result, it was able to accurately separate different lesion regions in 3D medical images^15^. Researchers used the modified seeded region growing algorithm to develop a semi-automated model for measuring and segmenting clots. The outcomes revealed better performance as compared to the traditional approaches^16^. Suberi Anis Azwani Muhd, et al. presented a computer-aided approach (CAD) for early DVT diagnosis. The system has improved the image quality using image processing techniques such as enhancement, segmentation, and morphology^17^. A deep neural network (DNN)-based CAD system was developed to segment and classify DVT and evaluate the DL approaches. The system was segmented with 2D U-Net, 2D VGG, and 3D U-Net and classified using 2D ResNet, CNN- recurrent neural network (RNN), and 3D Inception. VGG outperformed CNN-RNN without masks in segmentation, whereas 3D Inception with masks performed best in classification^18^. The researchers have used the mask R-CNN DNN, which was trained on PE images. The performance of their model was manually evaluated and compared with existing methods. It was stated that their model exhibited high performance in identifying locations and the size of the PE^19^. Table-1 presents a comparative analysis of various relevant approaches.

**Table 1:** A comparative studies of different pertinent approaches.

Mechanism	Features	Advantages	Limitations	Observations	Key Finding	Effectiveness in Segmentation Tasks
CNN ^7^	Fully automated segmentation	High accuracy, fully automated	Potential overfitting	Effective for lower extremity DVT	CNN effective for automated segmentation	High
Comparison of DL and ML methods ^8^	Classification of iliofemoral DVT on CT venography	Comparison provides insights on performance	Resource-intensive comparison	DL methods outperform conventional ML	DL shows superior performance	High (DL methods)
CNN with light reflection rheography^9^	Detection with light reflection rheography	Non-invasive detection	Dependent on quality of light reflection	Promising for non-invasive detection	CNN effective with rheography	Moderate
U-Net enhanced class activation map^10^	Multi-output network, robust classification	Enhanced performance with U-Net	Complexity, high computational cost	Robust against varied data	U-Net enhances classification performance	High
Multiscale CNN ^11^	Deep multiscale convolutional network	Handles multiscale features	Complex model training	Handles complex segmentation tasks	Multiscale CNN effective for medical segmentation	Significant
Attention-guided U-Net with Transformer^12^	Multi-level attention-guided segmentation	Combines attention mechanisms with transformer	Complexity, resource-intensive	Improved segmentation accuracy	Attention mechanisms improve accuracy	Considerable
Multi-scale attention net (MA-Net)^13^	Background-foreground segmentation	Effective background-foreground separation	Requires extensive training data	Clear separation of features	MA-Net effective for segmentation	Potential
3D DL^18^	3D analysis of lower extremity CT	Detailed 3D screening	High computational cost	Detailed analysis possible	3D DL effective for DVT screening	Substantial
Advanced Imaging Techniques^20^	Advanced imaging in acute and chronic DVT	Comprehensive imaging insights	Requires advanced imaging equipment	Useful for both acute and chronic cases	Advanced imaging provides comprehensive analysis	Moderate to High

Dang Truong, et al. provided a two-layer ensemble of DL models for segmenting medical images, which included probability prediction and weight-based approaches. Extension to image classification tasks, ensemble selection for optimal subsets, and parallelization of cross-validation processes were among the next improvements^21^. The U-Net segmentation model improved organ and lesion segmentation accuracy by incorporating different scale semantics of feature maps, directing model pruning, and attaining higher accuracy using two classification-guided modules^22^. Researchers introduced AdaResU-Net, which was based on adaptive CNN designed specifically for medical image segmentation. This network was capable of automatically adjusting to inputs and also reduced the network size^23^. New technologies have been leading to the development of different imaging modalities. A study by de Jong et al. explored potential applications of AI in PE diagnosis^24^. Karande Gita Yashwantrao, et al. established methods for the identification and diagnosis of DVT investigating promising innovative techniques and recent trends in this field^20^. The study carried out by Hemalakshmi G. R, et al. introduced attention-based multi-task model (Y-Net) for PE segmentation and detection. This model, combined with multi head attention mechanism, enhanced performance by concentrating on significant regions and minimizing irrelevant information^25^. Metlek Sedat proposed CellSegUNet approach for image segmentation based on U-Net++ and residual blocks, and it features a redesigned encoder block structure compared to traditional models^26^.

### Highlights of gap in the literature

1. The most significant issue accentuated in the studies is over-segmentation. Class imbalances in DVT analysis datasets frequently impact model performance and training. Model consistency is also impacted by image quality and variability, which calls for preprocessing actions like contrast and normalization. Model development is hindered by a lack of labeled data. Conventional qualitative evaluation is tedious and is susceptible to misinterpretation.
2. The erratic architecture of the examined literature makes automated analysis highly challenging, resulting in increased noise sensitivity.
3. The paucity of labelled data for training has an impact on the performance of DL models.
4. Manual annotating of data is cost-intensive and difficult to execute. As a result, self-supervised and transfer learning techniques need to be analysed and implemented.

### Significant limitations of these papers are as follows:

- The majority of DVT-based research has employed baseline models, which include conventional ML approaches or well-known DL architectures like basic CNNs or random forests, as a starting point for comparison. These models are generally used as benchmarks to assess the effectiveness of more sophisticated or tailored methods for diagnosing DVT.
- DVT analysis datasets often have a significant imbalance between positive and negative cases, affecting model training and performance. Image quality and variability also affect model consistency, requiring different preprocessing steps like normalization and contrast adjustment. DVT datasets often lack sufficient annotated data for segmentation tasks, limiting the development and validation of accurate models.
- The focus is on diagnosing DVT, without providing knowledge on its various categories or techniques for addressing and segmenting it.
- The specific scenario of DVT syndrome is not addressed in any scientific research investigations and recommendations.
- The variability in imaging modalities (CT, MR) presents challenges in developing models that generalize well across different datasets. We now specify how this impacts cross-study comparisons and results. Many DL models are black-box systems, which poses a challenge in clinical validation and trust. This lack of interpretability limits the adoption of these models in practice.

The authors aim to overcome the limitations of this research, with the following highlights being summarised:

- The paper presents theoretical knowledge on DVT and its risk factors, enabling the development of techniques for segmenting DVT.
- It analyses available datasets, provides a proposed framework and architecture that involves the baseline of the algorithm and develops an advanced method for medical image segmentation and analyses the performance of the model.
- Our research proposes DL algorithms for diagnosing and segmenting DVT and offers advanced research directions for DVT diagnosis and classification.

## Proposed methodology

**Fig. 2:**
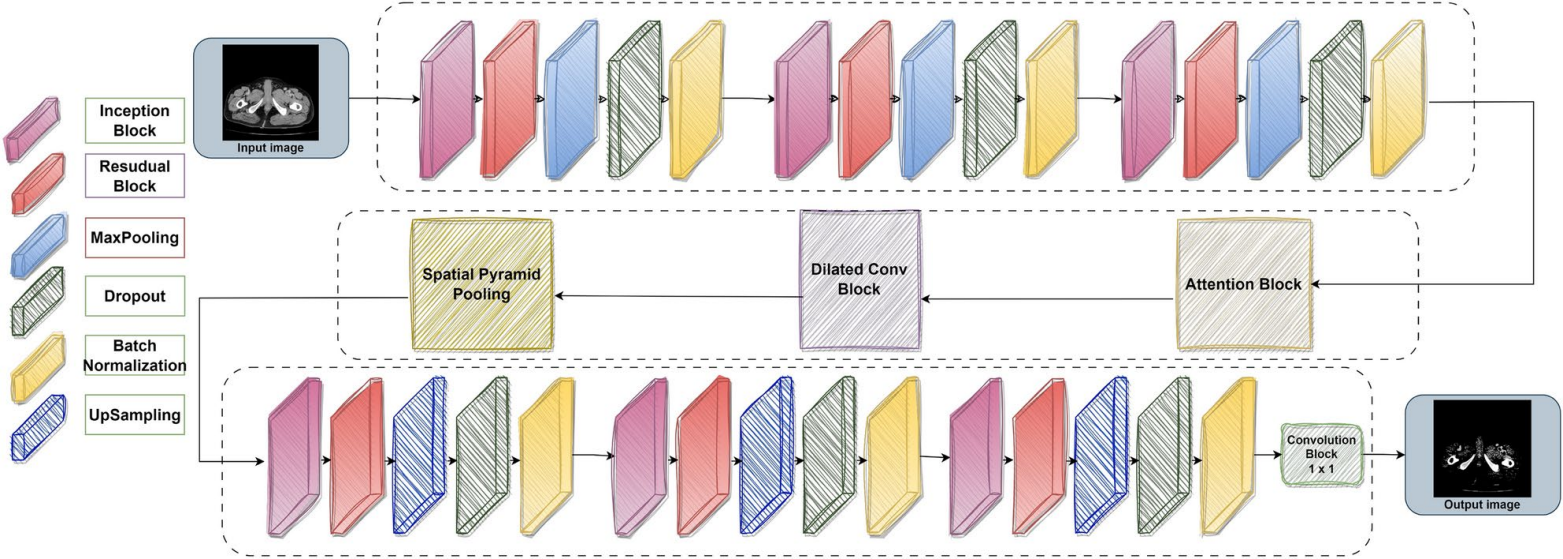
The workflow of modified-Net architecture model.

Our research is to design and build a Modified-Net architecture with attention mechanisms for DVT segmentation. We are using publicly available CT venography data, specifically the axial view of the left lower limb DVT, as our input dataset^27^. The preprocessing stage enhances the quality of images by boosting contrast, reducing noise, normalizing intensity, ensuring uniform input, and assisting in feature extraction. The preprocessing stage uses techniques like adaptive histogram equalization, noise reduction, intensity normalization, uniform input size, and feature extraction assistance to improve image quality and ensure dataset consistency. These techniques enhance contrast, minimize noise, normalize image intensities, and emphasize important structures for accurate feature extraction during the learning process. The heart of our framework is the modified-Net architecture, specifically designed for medical image segmentation. The encoder uses a variety of specialized blocks, including inception, spatial pyramid pooling, dilated convolution, attention, and residual blocks. These blocks extract hierarchical features, capturing intricate patterns and variations at multiple resolutions. An attention mechanism is integrated into the network’s intermediate stage to enhance feature representation. It calculates attention maps that emphasize relevant areas and suppress irrelevant ones, thereby improving the model’s segmentation precision. In the decoder section, the network employs up-sampling layers to revert the segmented regions to the original image size. Each decoder block refines the segmented regions, enhancing the spatial resolution of the segmentation masks by reconstructing details. The output layer of our model generates a probability map, where each pixel’s value indicates the likelihood of belonging to the specified class. We apply a threshold to this probability map to create the final binary segmentation mask, which classifies each pixel as either part of the region of interest or the background. In the training phase, our model reduces the discrepancy between the predicted segmentation mask and the ground truth annotations using binary cross-entropy loss and the Adam optimizer. In the testing phase, the trained model creates a segmentation mask for DVT regions from an input image. This mask can be further refined or directly used for medical diagnosis and treatment planning. By incorporating different architectural blocks, our modified-Net approach demonstrates enhanced performance in segmenting DVT regions. This robust technique helps medical professionals make precise diagnoses and treatment decisions, ultimately enhancing patient care and outcomes. Proposed model is an integration of convolutional layers, Residual, Inception, Dilated Convolution, Spatial Pyramid Pooling, and Attention blocks within the modified-Net architecture as shown in Figure-2.

### Residual block

Residual Block plays a key role in learning residual mappings, which is beneficial for deeper networks. It consists of two convolutional layers with a rectified linear unit (ReLU) as an activation function, followed by batch normalization and dropout regularization. This setup allows our model to effectually capture and study residual features, which is essential for deeper networks. The residual connection, attained through the addition operation between the input and output of the second convolutional layer, enables the network to mitigate the vanishing gradient problem and facilitates the training of deeper architectures.

The purpose of this block, based on the ResNet architecture, is to help mitigate the vanishing gradient problem by introducing skip connections. It allows the gradient to flow directly through the network, making it easier to train deeper networks. We contribute it to improve the gradient flow, which enables the training of deeper networks and can also potentially capture more complex patterns and representations.

Output of the residual block, denoted as ${\textbf {X}}_{\text {residual}}$, is obtained by adding the input tensor ${\textbf {X}}$ to the result of applying ReLU activation, dropout, and batch normalization to the output of a convolutional operation with filters ${\textbf {F}}$, kernel size ${\textbf {K}}$, and padding ${\textbf {P}}$.

**Output:**

$${\textbf {X}}_{\text {residual}} = {\textbf {X}} + {\textbf {H}}({\textbf {B}}({\textbf {D}}(\text {Conv2D}({\textbf {X}}, {\textbf {F}}, {\textbf {K}}, {\textbf {P}}))))$$

### Inception block

Inception Block, inspired by Google’s Inception module, integrates features from multiple receptive fields. Comprising convolutional layers with varying kernel sizes (1x1, 3x3, 5x5), alongside a max-pooling layer, this block enables the network to capture features at different spatial scales effectively. By using information from diverse receptive fields, the model becomes more robust to variations in object sizes and shapes present in the input images, enhancing its segmentation capabilities. The contribution enhances the model’s ability to extract features at various scales, capturing both fine and coarse details in the input image.

Output of the inception block, denoted as ${\textbf {X}}_{\text {inception}}$, is computed by concatenating the results of applying convolutional operations with different kernel sizes ${\textbf {K}}_i$ and a max pooling layer to the input tensor ${\textbf {X}}$, followed by ReLU activation.

**Output:**

$${\textbf {X}}_{\text {inception}} = {\textbf {H}}([\text {Conv2D}({\textbf {X}}, {\textbf {F}}, {\textbf {K}}_1, {\textbf {P}}), \text {Conv2D}({\textbf {X}}, {\textbf {F}}, {\textbf {K}}_2, {\textbf {P}}),\text {Conv2D}({\textbf {X}}, {\textbf {F}}, {\textbf {K}}_3, {\textbf {P}}), {\textbf {M}}({\textbf {X}})])$$

### Dilated convolution block

In Dilated Convolution Block dilated convolutions happen, which expands the receptive field of the network without losing the spatial resolution. It is also known as atrous convolutions. Through applying convolutions with holes (dilation rate > 1), this effectively captures contextual data from a broader area while preserving finer details. This feature is mainly helpful for medical image segmentation tasks, where precise delineation of structures is crucial.

The purpose of incorporating these blocks into our model is to increase the receptive field without reducing resolution. They help capture larger contextual information without downsampling the feature map. The model incorporates dilated convolutions, allowing it to consider a wider context, making it particularly useful for segmentation tasks requiring contextual information.

Output of the dilated convolution block, denoted as ${\textbf {X}}_{\text {dilated}}$, is obtained by applying a convolutional operation with filters ${\textbf {F}}$, kernel size ${\textbf {K}}$, and a specified dilation rate ${\textbf {D}}$ to the input tensor ${\textbf {X}}$.

**Output:**

$${\textbf {X}}_{\text {dilated}} = \text {Conv2D}({\textbf {X}}, {\textbf {F}}, {\textbf {K}}, \text {padding='same'}, \text {dilation\_rate}={\textbf {D}})$$

### Spatial pyramid pooling block

Spatial Pyramid Pooling Block helps to capture features at multiple scales without introducing additional parameters. By performing global max pooling, global average pooling, and 1x1 convolutions followed by global average pooling, this block aggregates information across different spatial dimensions. Consequently, the model becomes capable of handling objects of varying sizes within the input images, contributing to its versatility and robustness.

The purpose of this block is to capture information at multiple scales by using different pooling sizes. This is beneficial for handling objects of various sizes in the input image. The model enhances its ability to make predictions at various scales, making it more robust to variations in object sizes and improving overall segmentation performance.

Output of the spatial pyramid pooling, denoted as ${\textbf {X}}_{\text {pooling}}$, is formed by concatenating the results of global max pooling, global average pooling, and another global average pooling operation applied to the input tensor ${\textbf {X}}$, followed by ReLU activation.

**Output:**

$${\textbf {X}}_{\text {pooling}} = [{\textbf {M}}({\textbf {X}}), {\textbf {A}}({\textbf {X}}), {\textbf {A}}(\text {Conv2D}({\textbf {X}}, {\textbf {F}}, (1, 1), \text {padding='same'}))]$$

### Attention block

Attention Block incorporates an attention mechanism to selectively focus on relevant regions of the input feature map. By comparing the input feature map with a guidance signal (often a feature map from a previous layer), the block generates attention maps that highlight informative regions while suppressing noise and irrelevant features. This attention mechanism enhances the discriminative power of the network, enabling it to allocate more resources to crucial areas during the segmentation process.

The purpose of the attention mechanisms is to focus on relevant parts of the input while suppressing irrelevant regions. In this case, it helps the model focus on important features during the segmentation task. Our contribution to the attention block enhances the model’s ability to attend to critical regions, potentially improving the delineation of DVT related structures and reducing false positives.

Output of the attention block, denoted as ${\textbf {X}}_{\text {attention}}$, is computed by applying ReLU activation to the sum of the input tensor ${\textbf {X}}$ and a gating tensor ${\textbf {G}}$, followed by a sigmoid activation applied to the result of a convolutional operation with filters ${\textbf {F}}$ and kernel size ${\textbf {K}}$, resulting in an attention mask ${\textbf {F'}}$ which is then element-wise multiplied with the input tensor ${\textbf {X}}$.

**Output:**

$${\textbf {F}} = {\textbf {H}}({\textbf {X}} + {\textbf {G}})$$

$${\textbf {F'}} = {\textbf {S}}(\text {Conv2D}({\textbf {F}}, {\textbf {F}}, (1, 1), \text {padding='same'}))$$

$${\textbf {X}}_{\text {attention}} = {\textbf {X}} \cdot {\textbf {F'}}$$

These blocks collectively contribute to the model’s ability to capture intricate details, to handle variations in object sizes, and to focus on relevant features. In essence, the integration of specific modules in our modified-Net structure equips the model with the ability to proficiently delineate regions. The model’s improved performance and stability, which contribute to more precise and dependable medical image interpretation and diagnosis, are achieved by utilizing residual links, extracting features at multiple scales, employing dilated convolutions, implementing spatial pyramid pooling, and applying attention mechanisms.

The proposed segmentation technique for diagnosing DVT from CT venography images improves early diagnoses, reduces complications, and aids physicians in creating personalized treatment plans. It also enhances monitoring, reduces diagnostic workload, and can be extended to other vascular diseases, highlighting the importance of integrating advanced ML techniques in clinical workflows.

## Algorithm

**Algorithm:** Modified-Net Framework.

**Input:** Input image tensor with masks.

**Output:** Predicted segmentation which segments DVT.

**Steps:**

- **Initialization:** Provide the Modified-Net architecture incorporating the output layer, dilated convolution, encoder, intermediate block with attention, and spatial pyramid pooling.
- **Encoder:** Apply a residual block to the input after applying the inception block. Utilize max-pooling to down-sample feature maps, then dropout and batch normalization.
- **Intermediate block with attention mechanism:** Connect the encoder’s output to an inception block. Compute the attention mechanism by utilizing convolutional layers to combine the encoder’s output with the input feature maps. Sigmoid stimulation can be used to normalize attention weights. Using the encoder’s output, multiply the attention weights to get attended feature maps.
- **Dilated convolution block:** On the attended feature maps, apply dilated convolutions with a dilation rate of two.
- **Spatial pyramid pooling:** To extract multi-scale data from feature maps, use spatial pyramid pooling. Combine 1x1 feature map convolutions with global max-pooling.
- **Decoder:** Transpose convolutions are used to up-sample feature maps. Apply inception and residual blocks to every feature map that has been up-sampled. Up-sample feature maps successively until the desired output size is reached.
- **Output layer:** To get the final segmentation mask output, apply a convolutional layer with sigmoid activation.
- **Model compilation:** Use the Adam optimizer with a binary cross-entropy loss and a learning rate of 0.001 to compile the model.
- **Training:** Use labeled data and segmentation masks based on ground truth to train the model.
- **Prediction:** To forecast segmentation masks for fresh input images, apply the trained model.

## Results and interpretation

**Fig. 3:**
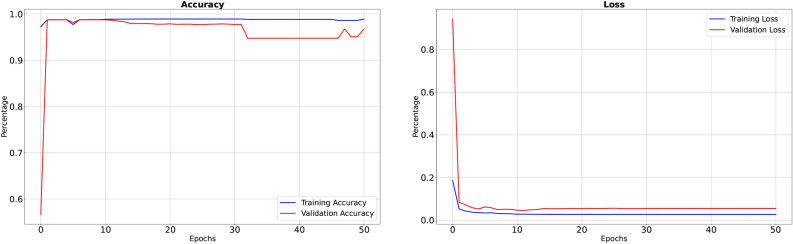
Graphical representation of performance metric: accuracy and loss.

**Fig. 4:**
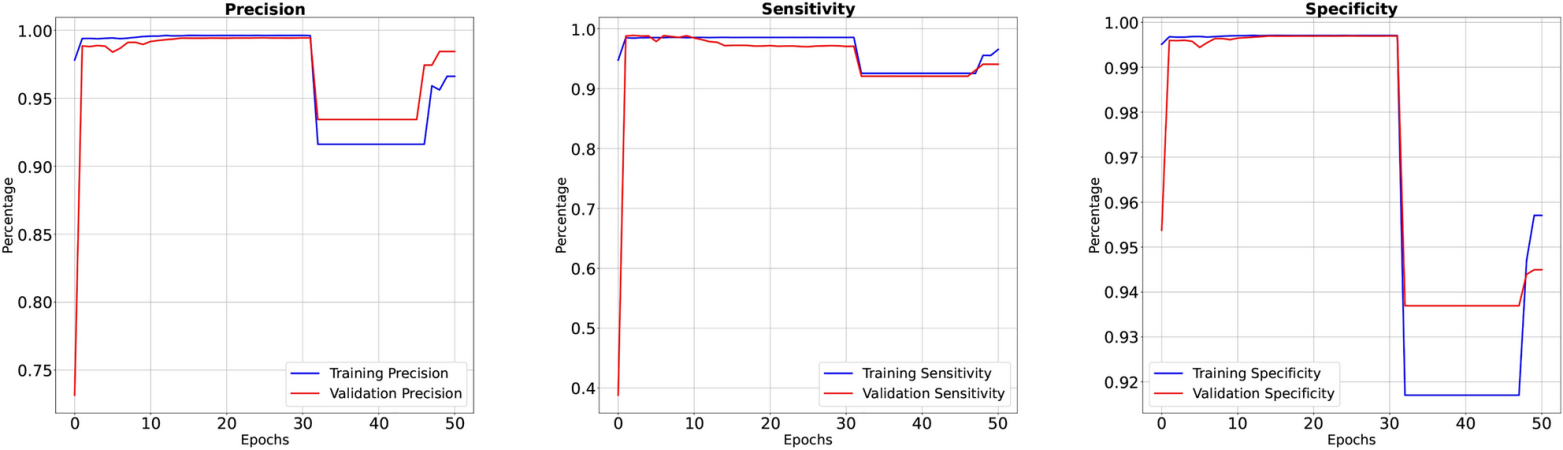
Graphical representation of performance metric: precision, sensitivity, and specificity.

Our Modified-Net Model, enriched with a combination of advanced architectural blocks made-to-order for DVT segmentation, demonstrates remarkable performance across various metrics. It also addresses the challenges in DVT segmentation tasks by utilizing residual blocks, inception blocks, dilated convolutions, spatial pyramid pooling, and attention mechanisms. After training the model using the Adam optimizer with a learning rate of 0.001 for 50 epochs on an HP system equipped with an Intel Core i7-1165G7 processor and 64GB of RAM running Windows 11, we achieved compelling results. The training phase yielded an accuracy of 98.92% with a corresponding loss of 0.02692. Furthermore, precision, recall, sensitivity, specificity, dice and IoU metrics were observed as 98.61%, 95.48%, 96.55%, 96.70%, 97.48% and 95.10% respectively. During validation phase, our model demonstrated a validation accuracy of 96.77%, accompanied by a validation loss of 0.05487. Precision, recall, sensitivity, specificity, dice and IoU were measured at 97.43%, 96.14%, 94.07%, 94.49%, 95.65% and 91.63% respectively. The graphical representations of accuracy and loss can be observed in Figure-3, while Figure-4 depicts precision, sensitivity, and specificity. These results underscore the efficacy of our proposed framework in accurately delineating DVT regions, highlighting its potential for clinical application and further research exploration.

### Performance evaluation

We have listed the important parameters used for model training and evaluation of our model in Table 2 in order to promote transparency and guarantee reproducibility of our training setup. Important details including the optimizer, learning rate, data partitioning plan, and other hyperparameters like batch size and early stopping conditions are listed in this table. We provide a clear understanding of the methods employed in our experiments by providing these facts.

**Table 2:** Training Configuration of our Modified-Net.

Parameter	**Description**
Model	Modified-net
Data partitioning	Random split (80% train, 10% validation, 10% test)
Optimizer	Adam
Learning rate	0.001
Activation function	ReLU
Loss function	Binary cross-entropy
Number of epochs	50
Batch size	16
Early stopping	Patience of 10 epochs (based on validation loss)
Dropout rate	0.25
Batch normalization	Applied after key layers to stabilize and accelerate training

### Quantitative analysis metrics

The performance of our model is assessed using several evaluation metrics, which provide insights into its effectiveness in segmenting DVT. Following are the metrics used in this study are given in Table 3.

**Table 3:** Evaluation metrics with formulas and its significance.

Metric	Formula	Meaning
Accuracy	$\frac{P_{\text {correct}} + N_{\text {correct}}}{P_{\text {correct}} + N_{\text {correct}} + P_{\text {incorrect}} + N_{\text {incorrect}}}$	Compares the number of accurate predictions-both positive and negative against the total number of predictions in order to assess the overall accuracy of the model.
Precision	$\frac{P_{\text {correct}}}{P_{\text {correct}} + P_{\text {incorrect}}}$	Demonstrates the model’s capacity to reduce false positives by displaying the proportion of true positive predictions among all positive predictions.
Sensitivity	$\frac{P_{\text {correct}}}{P_{\text {correct}} + N_{\text {incorrect}}}$	Demonstrates the efficacy of the model in detecting DVT by accurately identifying true positives among all actual positive cases.
Specificity	$\frac{N_{\text {correct}}}{N_{\text {correct}} + P_{\text {incorrect}}}$	Evaluates the model’s ability to prevent false positives by calculating the percentage of accurate negative predictions across all real negative cases.
Loss	$-\frac{1}{N} \sum _{i=1}^{N} [y_i \log (\hat{y}_i) + (1 - y_i) \log (1 - \hat{y}_i)]$	Calculates the prediction error of the model and uses the difference between the expected and actual values to optimize the model.
Dice	$\frac{2P_{\text {correct}}}{2P_{\text {correct}} + P_{\text {incorrect}} + N_{\text {incorrect}}}$	Measures the overlap between the predicted positive class and the actual positive class, assessing segmentation accuracy in terms of overlap between the two sets.
Intersection over Union (IoU)	$\frac{P_{\text {correct}}}{P_{\text {correct}} + P_{\text {incorrect}} + N_{\text {incorrect}}}$	Calculates the intersection over union of the predicted and actual segments, reflecting the proportion of overlap between the prediction and ground truth.

The binary cross-entropy loss measures the difference between the predicted probabilities and the actual labels, guiding the model’s optimization during training.

Where $P_{\text {correct}}$ refers to True Positives, $N_{\text {correct}}$ represents True Negatives, $P_{\text {incorrect}}$ stands for False Positives, and $N_{\text {incorrect}}$ corresponds to False Negatives. Additionally, $y_i$ denotes the actual labels, $\hat{y}_i$ the predicted probabilities, and $N$ the total number of samples.

These metrics provide a comprehensive evaluation of the model’s performance, facilitating comparisons with other existing techniques in DVT segmentation. Our obtained results are displayed in a tabular format in Table-4.

**Table 4:** Performance evaluation of our proposed framework Modified-Net.

Parameters	Training Set	Validation Set
Accuracy	98.92%	96.77%
Loss	0.02692	0.05487
Precision	98.61%	97.43%
Dice	97.48%	95.65%
IoU	95.10%	91.63%
Sensitivity	96.55%	94.07%
Specificity	96.70%	94.49%

**Fig. 5:**
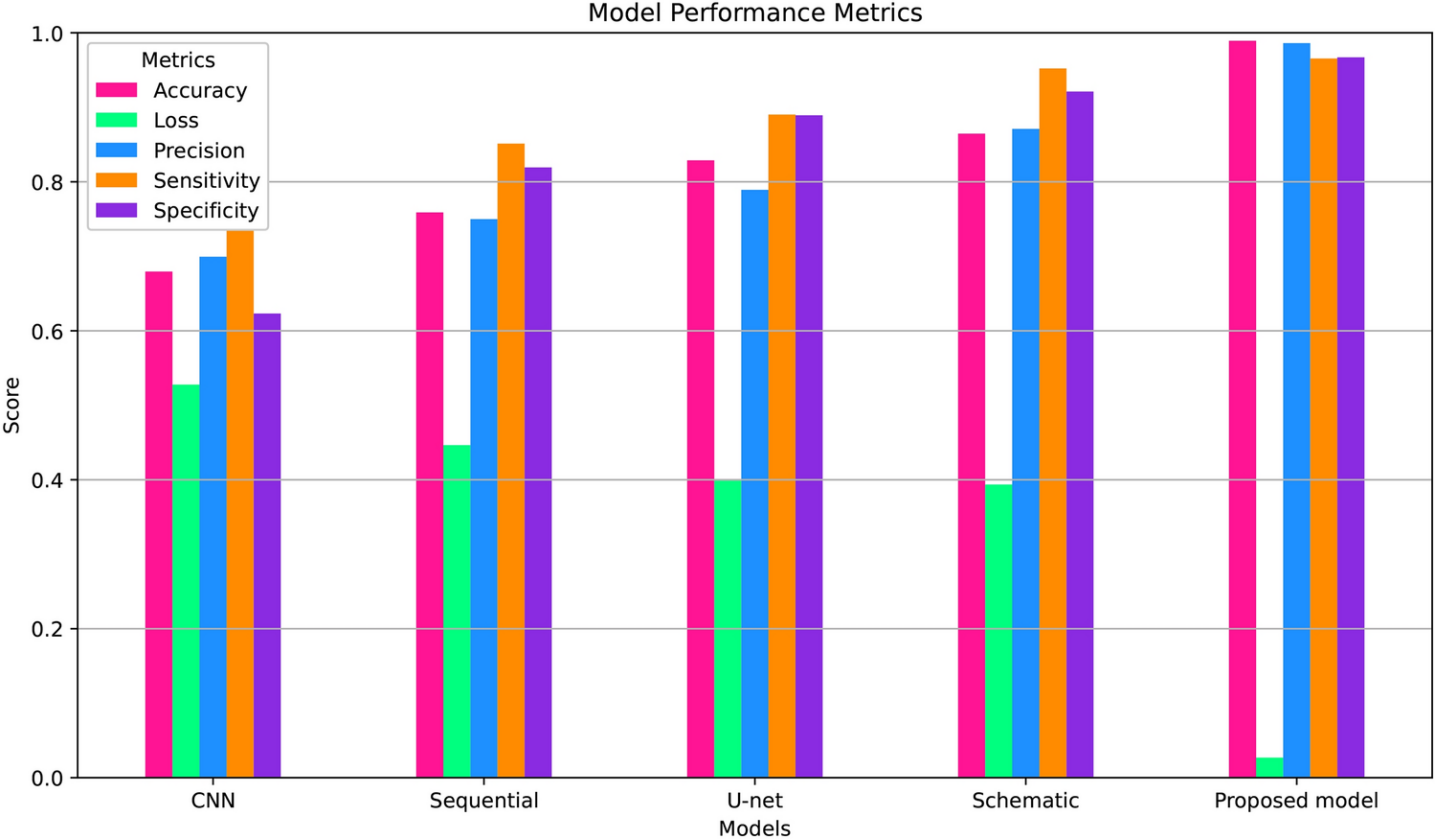
Comparative summary between our proposed model and traditional techniques.

### Comparison of recent studies

In our research, we examined and compared existing techniques such as CNN, U-Net, Sequential, Schematic, and our proposed Modified-Net. We evaluated each method’s performance metrics, including accuracy, loss, sensitivity, and precision. Our Modified-Net consistently surpassed and outperformed conventional methods. Figure-5 graphically represents the comparative outcomes. Compared results which was also trained and executed. The numerical values of outcomes are given in Table-5.

**Table 5:** Comparison of our performance with conventional techniques.

Model	Accuracy	Loss	Precision	Sensitivity	Specificity
CNN	0.6795	0.5277	0.6994	0.7527	0.623170
Sequential	0.7589	0.4465	0.7499	0.8512	0.819276
U-net	0.8288	0.3987	0.7892	0.8902	0.889290
Schematic	0.8646	0.3936	0.871	0.9521	0.921218
Our model	0. 989240	0.02692	0.986140	0.965536	0.967053

## Conclusion

Our novel modified-Net framework is specifically designed for the segmentation of DVT regions from medical images. Through the integration of advanced architectural block, including spatial pyramid pooling, dilated convolutions, residual blocks, inception blocks, and attention mechanisms, we have devised a methodical strategy to tackle the difficulties related to DVT segmentation. Our proposed framework outperforms conventional techniques in accurately defining DVT regions by exhibiting better segmentation performance when compared to conventional techniques.

## Discussion and future directions

Modified-Net framework gave promising outcomes, but further research is needed to improve segmentation robustness and precision. The effectiveness of the model could be improved by adding more architectural features, patient-specific data, multi-modal imaging data, and temporal information. Unsupervised or semi-supervised learning strategies could improve scalability and generalization. Our framework serves as a foundation for future research on DVT segmentation practices. Implementation in clinical settings and real-world studies are crucial for evaluating its effectiveness.

## System requirements

Employing the Google Colab platform, we successfully developed and executed Python scripts specifically designed for a ML application. The experimental setup was configured with a Windows 11 operating system, an 11th Gen Intel$\circledR$ Core$^\textrm{TM}$ i7-1195G7 processor operating between 2.90GHz and 2.92GHz, a 64-bit system architecture, and 16GB of RAM.

## Data Availability

All data subjected to analysis for this review are encapsulated within this published scholarly piece. The data employed and/or examined within the manuscript can be obtained from the corresponding author upon a justifiable request.
